# High expression of CXCL13 predicts a favorable response to immunotherapy by upregulating CXCR5+CD8+ T-cell infiltration in gastric cancer

**DOI:** 10.3389/fimmu.2025.1551259

**Published:** 2025-05-08

**Authors:** Shuning Xu, Danyang Li, Tao Ning, Yao Lu, Yansha Sun, Hua Bai, Lei Qiao, Ting Deng, Ying Liu

**Affiliations:** ^1^ Department of Medical Oncology, The Affiliated Cancer Hospital of Zhengzhou University, Henan Cancer Hospital, Zhengzhou, China; ^2^ Department of Gastrointestinal Medical Oncology, Tianjin Medical University Cancer Institute and Hospital, National Clinical Research Center for Cancer, Tianjin’s Clinical Research Center for Cancer, Tianjin Key Laboratory of Digestive Cancer, Key Laboratory of Cancer Prevention and Therapy, Tianjin, China; ^3^ Department of Medical Oncology, Zhengzhou People’s Hospital, Zhengzhou, China

**Keywords:** gastric cancer, immunotherapy, prognosis markers, CXCL13, CXCR5

## Abstract

**Introduction:**

Identifying predictive biomarkers for immune checkpoint inhibitor (ICI) treatment is critical for gastric cancer (GC) prognosis. C-X-C motif chemokine ligand 13(CXCL13) plays an important role in immune regulation by binding exclusively to its receptor CXCR5. However, its role, underlying mechanisms, and prognostic significance in ICI-treated GC patients remain controversial.

**Methods:**

This study investigated the clinical significance of CXCL13 and its potential immunomodulatory function in GC patients. A total of 144 GC patients from two cohorts, who received a combination of chemotherapy and anti-PD-1 antibody, were analyzed. The expression of CXCL13 was assessed using immunohistochemistry (IHC) and enzyme-linked immunosorbent assay. Associations between CXCL13, CXCR5, CD8, and CD4 were assessed by IHC and immunofluorescence. Survival analysis was performed using the Kaplan–Meier method and Cox proportional hazards model. The treatment response to CXCL13 and anti-PD-1 antibody was investigated using a subcutaneous xenograft tumor mouse model.

**Results:**

The results suggested that patients with high CXCL13 expression had prolonged survival. High CXCL13 expression exhibited increased infiltration of CXCR5+CD8+ T cells and was associated with better outcomes. The combined assessment of CXCL13, CXCR5, and CD8+ T cells served as an independent predictor of prognosis. Additionally, CXCR5 and CD8+ T cells were enriched in tertiary lymphoid structures (TLSs), which conferred a prognostic benefit in the presence of high CXCL13 expression. CXCL13, in combination with anti-PD-1 therapy, retarded tumor growth in vivo, resulting in increased infiltration of CXCR5+CD8+ T cells.

**Discussion:**

This study identified CXCL13 as a prognostic factor in GC patients receiving ICI therapy, emphasizing its critical role in the antitumor microenvironment via CXCR5+CD8+ T cells.

## Introduction

Gastric cancer (GC) ranks fifth in terms of incidence and fourth in mortality among cancers worldwide (1). Approximately 70% of patients in China are diagnosed with advanced or metastatic GC at initial presentation (2). Immune checkpoint inhibitors (ICIs) have transformed the treatment paradigm for advanced GC. The combination of anti-PD-1 antibodies and chemotherapy improves the survival of patients with advanced GC (3–5). Extensive research has been conducted on molecular markers associated with ICIs. In GC, HER-2 positivity (6), PD-L1 CPS ≥ 1 (7), microsatellite instability-high/mismatch repair protein deficiency (8, 9), tumor mutational burden-high (10), and Epstein–Barr virus(EBV) positivity (11) are good prognostic factors for immunotherapy. Unfortunately, the overall response rate of GC patients to immunotherapy remains below 15% (12). Consequently, the identification of biomarkers that predict immunotherapeutic responsiveness is of urgent importance.

The tumor microenvironment (TME) plays a crucial role in the immunotherapy response, particularly in individualized precision treatment strategies (13, 14). In our previous study, we assessed intratumoral transcriptomic changes in the TME at the single-cell level in GC patients receiving neoadjuvant anti-PD-1 antibody combined with mFOLFOX6. The results indicated that C-X-C motif chemokine ligand 13 (CXCL13)+CD8+ T cells were specifically enriched in responder patients (15). CXCL13 is a member of the chemokine CXC subfamily. Chemokines can be classified into CC, CXC, CX3C, and C subfamilies. They are predominantly 8- to 12-kDa secreted proteins that regulate directed cell migration (chemotaxis), adhesion, cell positioning, and cell–cell interactions by binding to chemokine receptors (16). In the TME, chemokines regulate immune cell trafficking and exert both pro- and antitumorigenic functions (17–19).

CXCL13 exerts antitumor effects by binding exclusively to the C-X-C motif chemokine receptor 5 (CXCR5) (20, 21) and plays a critical role in immune responses. Accumulating evidence has demonstrated that the CXCL13–CXCR5 axis significantly influences immune cell infiltration in the TME. Subpopulations of CD8-CXCL13 and CD4-CXCL13 T cells were significantly increased in liver metastatic samples of colon cancer, which exhibited high proliferative ability and tumor-activating characteristics, contributing to a better prognosis for patients (22). In ovarian cancer (23), high CXCL13 expression is associated with prolonged survival by shaping the anti-TME by facilitating the maintenance of CXCR5+CD8+ T cells. Nevertheless, the effects of CXCL13–CXCR5 maintenance on the TME of GC and its prognostic significance in ICI treatment remain unclear.

In this study, we aimed to elucidate the prognostic significance of CXCL13 and its functional association with CXCR5-expressing immune cells in GC patients. High expression of CXCL13 was associated with prolonged survival, and CXCL13 exerted its antitumor activity by recruiting CXCR5+CD8+ T cells in GC patients. Therefore, CXCL13 may serve as a valuable prognostic indicator and a potential therapeutic target for ICI treatment of GC.

## Materials and methods

### Patients and specimen collection

This study was conducted on two independent cohorts of GC patients from Tianjin Medical University Cancer Institute and Hospital and The Affiliated Cancer Hospital of Zhengzhou University. Formalin-fixed paraffin-embedded (FFPE) tumor tissues collected prior to ICI treatment from cohort 1 were used for immunohistochemical (IHC) and immunofluorescence staining. Serum samples were obtained from peripheral blood pre- and post-treatment (8–9 weeks following the first treatment) of patients in cohort 2. The selected patients received anti-PD-1 antibody combined with chemotherapy as a neoadjuvant or first-line treatment between 2020 and 2023. None of the enrolled patients had an autoimmune disease or a history of cancer, and none had received immunotherapy, chemotherapy, radiation, or any other antitumor therapy prior to the initiation of neoadjuvant or first-line treatment. Progression-free survival (PFS) was calculated from the first dose of first-line treatment to disease relapse, progression, or death. Overall survival (OS) was calculated from the first dose of first-line treatment to all-cause death or the last follow-up. Treatment responses were evaluated using the Response Evaluation Criteria in Solid Tumors version 1.1 (RECIST 1.1) and the College of American Pathologists Tumor Regression Grading System. Informed consent was obtained from all patients.

### IHC staining

FFPE specimens from pre-treatment tumor tissues in cohort 1 were prepared for IHC analysis. The following primary antibodies were used: CXCL13 (dilution 1:500; Abcam), CD4 (dilution 1:1200; Abcam, USA), CD8 (working solution; Zhongshan Jinqiao, China), CD20 (dilution 1:300; Invitrogen, USA), and CXCR5 (dilution 1:200; CST, USA). A complete list of antibodies is available in **Supplementary Table S1**. Normal lymph node tissue was used as a positive control, while an isotype antibody, instead of the primary antibody, served as the negative control. IHC results were independently evaluated by two pathologists who were blinded to the patient’s clinical data. CXCL13 and CXCR5 expression levels were scored by combining the proportion of positively stained cells with staining intensity. Staining intensity was graded on a scale of 0 to 3 (absent = 0, weak = 1, moderate = 2, and strong = 3), and the percentage of positive cells (range, 0%–100%) was determined using the Image J (NIH) algorithm. The average CXCL13 or CXCR5 H-score (range, 0–300) was calculated using the following formula: % positive cells × staining intensity, across five randomly selected high-power fields (HPFs) (×400). CD4 and CD8 expression levels were evaluated based on the number of positively stained tumor-infiltrating lymphocytes. Each sample was examined under high magnification (×400) across five randomly selected fields and the average count was recorded. Median protein expression levels were used as the cut-off values for defining high- and low-level expression. The cut-off values were 25 for CXCL13, 40 for CXCR5, 15 cells/HPF for CD8, and 22 cells/HPF for CD4. Tertiary lymphoid structures (TLSs) were identified as organized dense lymphocyte aggregates in hematoxylin and eosin (HE)-stained samples, with simultaneous positive IHC staining for CD20 within these regions.

### Immunofluorescence staining

Immunofluorescence was performed on pre-treatment tumor samples from 22 patients in cohort 1. The slides were incubated with goat anti-human CXCR5, rabbit anti-human CD8, or rabbit anti-human CD4 antibodies. Horseradish peroxidase (HRP)-conjugated goat anti-rabbit IgG and AlexaFluor^®^488-conjugated goat anti-rabbit secondary antibodies were used. Endogenous peroxidase activity was blocked with 3% H_2_O_2_ at room temperature for 15 min. The slides were then incubated with a rabbit recombinant anti-CD8 or anti-CD4 alpha antibody overnight at 4°C, followed by incubation with an HRP-conjugated goat antirabbit IgG antibody at room temperature for 50 min in the dark. The slides were subsequently stained with the TSA-CY3 solution for 10 min in the dark. Thereafter, the slides were incubated with a second primary antibody (recombinant anti-CXCR5 alpha antibody) overnight at 4°C. The tissue was then covered with a secondary antibody (AlexaFluor^®^488-conjugated goat anti-rabbit IgG) and incubated at room temperature for 50 min in the dark. The nuclei were counterstained with DAPI, and fluorescence microscopy was used for detection and image acquisition.

### Enzyme-linked immunosorbent assay

Enzyme-linked immunosorbent assay (ELISA) was performed to detect the expression of CXCL13, interferon-gamma (IFN-γ), tumor necrosis factor-alpha (TNF-α), Granzyme B (GZMB), interleukin (IL)-17A, and IL-2 using a commercially available ELISA kit (Liankebio, China). All assays were performed according to the manufacturer’s instructions. Cytokine concentrations were calculated based on standard curves generated using a specialized program for ELISA results evaluation.

### Public database analysis

The Cancer Genome Atlas (TCGA) GC and GSE66229 transcriptome profiles were downloaded from TCGA Data Portal (http://tcga-data.nci.nih.gov/tcga/) and the NCBI public data platform, respectively. Comparisons between tumor and normal samples were performed using the Wilcoxon rank-sum test, and comparisons between different tumor stages were conducted using nonparametric Kruskal–Wallis tests. The Kaplan–Meier survival analysis was performed to compare the prognoses of the two datasets.

### Animal experiments

#### Preparation of peripheral blood mononuclear cells

Fresh whole blood from healthy adults was collected using EDTA tubes. Peripheral blood mononuclear cells (PBMCs) were isolated using Ficoll lymphocyte gradient separation at 400×*g* for 30 min without acceleration. The second layer of the column was collected and washed with phosphate-buffered saline (PBS). After washing, the concentration of PBMCs was measured and adjusted to 1.0 × 10^7^ cells/ml. PBMCs were stored in a liquid nitrogen tank until use.

#### PBMC injection process in M-NSG mice

The animal experiments complied with ethical standards and were approved by the Ethical Committee of Zhengzhou University(2023-YYY-044). Female M-NSG is the a mouse strain, and the full name of the mice of this strain is NOD.Cg-Prkdc^scid^Il2rg^em1Smoc^. Mice were obtained from Shanghai Model Organisms Center Inc. and were used at 4 weeks of age. Mice were maintained under specific pathogen-free (SPF) conditions according to the SPF guidelines (room temperature, 40%–60% humidity). Experiments were initiated after 1 week of adaptive feeding. PBMCs from healthy adults were removed from liquid nitrogen storage and immediately thawed in a 37°C water bath. The PBMCs were then washed once with Hank's Balanced Salt Solution (HBSS) (Gibco, USA) and resuspended by mixing 1.0 × 10^7^ PBMCs with 200 μl of HBSS for intravenous injection into the tail vein of recipient M-NSG mice at 5 weeks of age (24, 25). CD45 expression in the peripheral blood was measured, and mice with over 25% CD45-positive cells were considered engrafted and humanized. Humanized M-NSG mice derived from different PBMC donors with varying engraftment levels were randomly assigned to each treatment group (**Supplementary Figure S1A**).

### GC cells for tumor engraftment

The human GC cell line MKN-28 was purchased from ATCC (ATCC; Manassas, VA, USA) and cultured in standard RPMI 1640 medium supplemented with 10% fetal bovine serum and antibiotics (100 U/ml penicillin and 100 µg/ml streptomycin) under standard culture conditions (5% CO_2_, 37°C). The MKN-28 cell line demonstrated high CXCR5 expression, as confirmed by polymerase chain reaction (PCR) analysis (**Supplementary Figure S1B**). The PCR primer sequences used were 5′-TCAGTGGGCCCTATGTAGGAA-3′ (upper strand) and 5′-TGATGGCCTTGGCTGACTTT-3′-(lower strand). Hu-PBMC-NSG mice were subcutaneously injected with 1.0 × 10^6^ MKN-28 cells under anesthesia at 6 weeks of age. The size of the subcutaneous xenograft tumors was measured every 3 days following injection. Tumor volume was calculated using the following formula: tumor volume = length × width × width/2.

### Tumor experiments

Treatment was initiated when the tumors reached a volume of 50–100 mm^3^. Hu-PBMC-CDX mice were randomly divided into four groups (control, CXCL13, anti-PD-1 antibody, and CXCL13 plus anti-PD-1 antibody), with each group containing six mice. Mice were administered peritumoral injections of CXCL13 (1.25 µg per mouse) every other day until the end of the experiment (26). Anti-PD-1 antibody (camrelizumab, Jiangsu Hengrui, China) was administered intraperitoneally (200 µg per mouse) every 3 days for a total of six doses. Mice in the control group received intraperitoneal PBS injection (200 µg per mouse) every 3 days. Mice were killed 2 days after the final anti-PD-1 antibody dose. Subcutaneous tumors, spleens, and peripheral blood were harvested and processed into single-cell suspensions for flow cytometry analysis.

### Flow cytometry

Single-cell suspensions were prepared from the subcutaneous tumors, spleens, and peripheral blood of Hu-PBMC-CDX-mice for flow cytometry. The cell suspensions were stained with a panel of fluorochrome-conjugated monoclonal antibodies (**Supplementary Table S1**). Staining was performed according to the manufacturer’s instructions (Beckman Coulter, USA). Flow cytometry was conducted using a FACS flow cytometer (Beckman Coulter), and data were analyzed using CytExpert (Beckman Coulter) and FlowJo software (Tree Star, USA).

### Statistical analysis

Data were analyzed using GraphPad Prism 9.0 and SPSS software (version 26.0). Differences between the two groups were assessed using an independent *t*-test. Statistically significant differences among multiple groups were determined by one-way analysis of variance (ANOVA) or two-way ANOVA. Differences were considered statistically significant at the following levels: ^*^ *p* < 0.05; ^**^ *p* < 0.01; ^***^ *p* < 0.001; and ^****^ *p* < 0.0001. Survival analysis was performed using the Kaplan–Meier method with the log-rank test, along with univariate and multivariate Cox proportional hazards models. A Chi-square test was conducted to assess the correlation between molecular marker expression and clinical features. Statistical significance was set at a *p*-value < 0.05.

## Results

### High expression of CXCL13 is associated with a good clinical response to immunotherapy in GC

A total of 144 patients were enrolled across two cohorts, with 89 patients in cohort 1 (between 2020 and 2022) and 55 patients in cohort 2 (between April 2023 and December 2023). All enrolled patients received anti-PD-1 antibodies combined with chemotherapy as first-line or neoadjuvant treatment. The median age was 63 years (range: 28–82) in cohort 1 and 66 years (range: 32–78) in cohort 2. Among the patients in cohort 1, 69 (77.5%) received platinum-based chemotherapy, and seven (7.9%) received taxane-based chemotherapy. Among cohort 2 patients, 53 (96.4%) received platinum-based chemotherapy, and one (1.8%) received taxane-based chemotherapy. The baseline clinical characteristics of the two cohorts are presented in **Table 1**.

**Table 1:** Patient characteristics of the two clinical cohorts.

	Cohort 1	Cohort 2
Variables	Total (*n* = 89)	Total (*n* = 55)
Median age (years; range)	63 (28–82)	66 (32–78)
Sex (*n*; %)		
Male	61 (68.5)	39 (71.0)
Female	28 (31.5)	16 (29.0)
HER-2 status (*n*; %)		
IHC 3+/FISH amplification	22 (24.7)	9 (16.3)
IHC 0–2+/FISH nonamplification	63 (70.8)	39 (71.0)
Uknow	4 (4.5)	7 (12.7)
MMR status (*n*; %)		
dMMR/MSI-H	2 (2.2)	2 (3.6)
pMMR/MSS	46 (51.7)	37 (67.3)
Uknow	4 1(46.1)	16 (29.1)
Histologic type (*n*; %)		
Adenocarcinoma	89 (100.0)	55 (100)
Signet-ring cell carcinoma	14 (15.7)	11 (20.0)
Primary tumor location (*n*; %)		
Gastroesophageal junction	23 (25.8)	29 (52.7)
Gastric	66 (74.2)	26 (47.3)
Tumor metastasis (*n*; %)		
Parenchyma organ	43 (48.3)	20 (36.4)
Nonparenchyma organ	46 (51.6)	19 (34.5)
No distant metastasis	N/A	16 (29.1)
Treatment therapy (*n*; %)		
Platinum-based	69 (77.5)	53 (96.4)
Taxane-based	7 (7.9)	1 (1.8)
Platinum-combined taxane-based	5 (5.6)	0 (0.0)
Others	8 (9.0)	1 (1.8)
Clinical stage		
III	N/A	16 (29.1)
IV	89 (100)	39 (70.9)
Efficacy (*n*; %)		
CR+PR	31 (34.8)	8 (14.5)
SD	51 (57.3)	38 (69.0)
PD	3 (3.4)	1 (1.8)
TRG 0–1	N/A	2 (3.6)
TRG 2–3	N/A	6 (10.9)
Uknow	4 (4.5)	0 (0.0)

**Figure 1:**
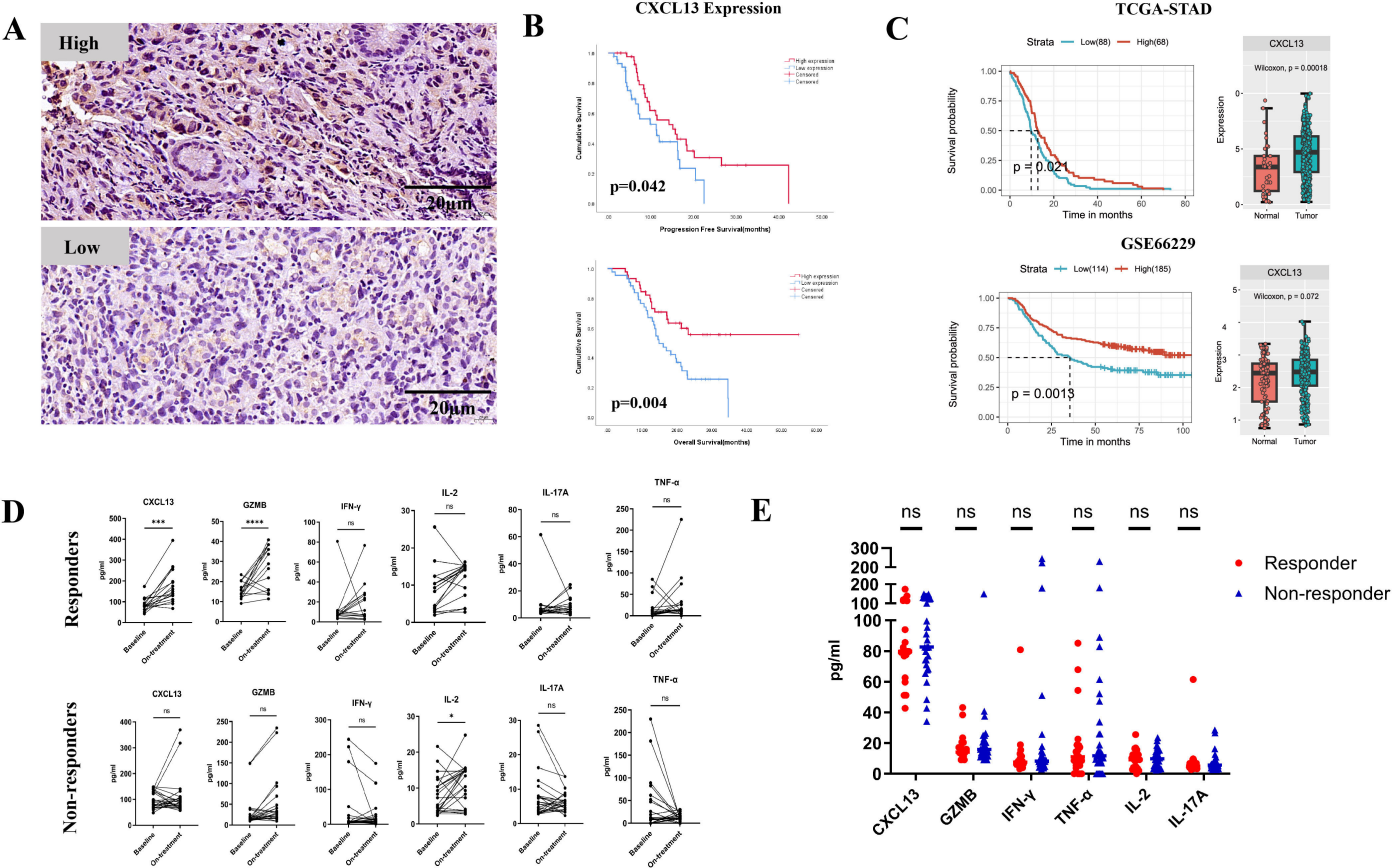
**(A)** Representative images showing high and low expression of CXCL13 by IHC staining. **(B)** Kaplan–Meier survival curves for PFS (top) and OS (bottom) in cohort 1 patients stratified by CXCL13 expression (*n* = 89; log-rank test, *p*-values shown). **(C)** Survival analysis of CXCL13 expression in TCGA and GEO datasets (left); expression of CXCL13 in tumor tissues vs. normal tissues (right). **(D)** Pre- and on-treatment (8–9 weeks from the first treatment) expression changes of CXCL13, GZMB, IFN-γ, IL-2, IL-17A, and TNF-α in serum samples between responders and non-responders in cohort 2 patients. **(E)** Baseline expression of CXCL13, GZMB, IFN-γ, IL-2, IL-17A, and TNF-α in responders and non-responders. (^*^ *p* < 0.05; ^***^ *p* < 0.001; ^****^ *p* < 0.0001; ns, not significant).

In cohort 1, patients were stratified into “high” and “low” expression groups based on the median IHC staining value for CXCL13 expression (**Figure 1A**). High CXCL13 expression was significantly correlated with longer PFS (median PFS: 20.0 months vs. 11.7 months, *p* = 0.042) and OS (median OS: 36.4 months vs. 18.7 months, *p* = 0.004) (**Figure 1B**). Furthermore, the evaluation of TGCA and GEO datasets confirmed that CXCL13 expression was higher in tumor tissues than in normal tissues. Upregulated CXCL13 expression was associated with prolonged survival in GC patients (**Figure 1C**).

In cohort 2, patients who achieved a complete response (CR), partial response (PR), or tumor regression grade (TRG) 0–1 were defined as the responder group (*n* = 21). Those with stable disease (SD), progressive disease (PD), or TRG 2–3 were defined as the non-responder group (*n* = 34). Among responders, CXCL13 expression was markedly elevated before treatment. Furthermore, an increase in the effector cytokine GZMB was observed in the responder group but not in the non-responders (**Figure 1D**). No statistically significant differences in baseline CXCL13 or effector cytokine expression levels were observed between responders and non-responders before treatment (**Figure 1E**). These results suggest that the upregulation of CXCL13 expression may enhance the efficacy and improve the survival outcomes of anti-PD-1 treatment in GC patients.

### CXCL13 expression was significantly correlated with intratumoral infiltration of CXCR5+CD8+ T cells

**Figure 2:**
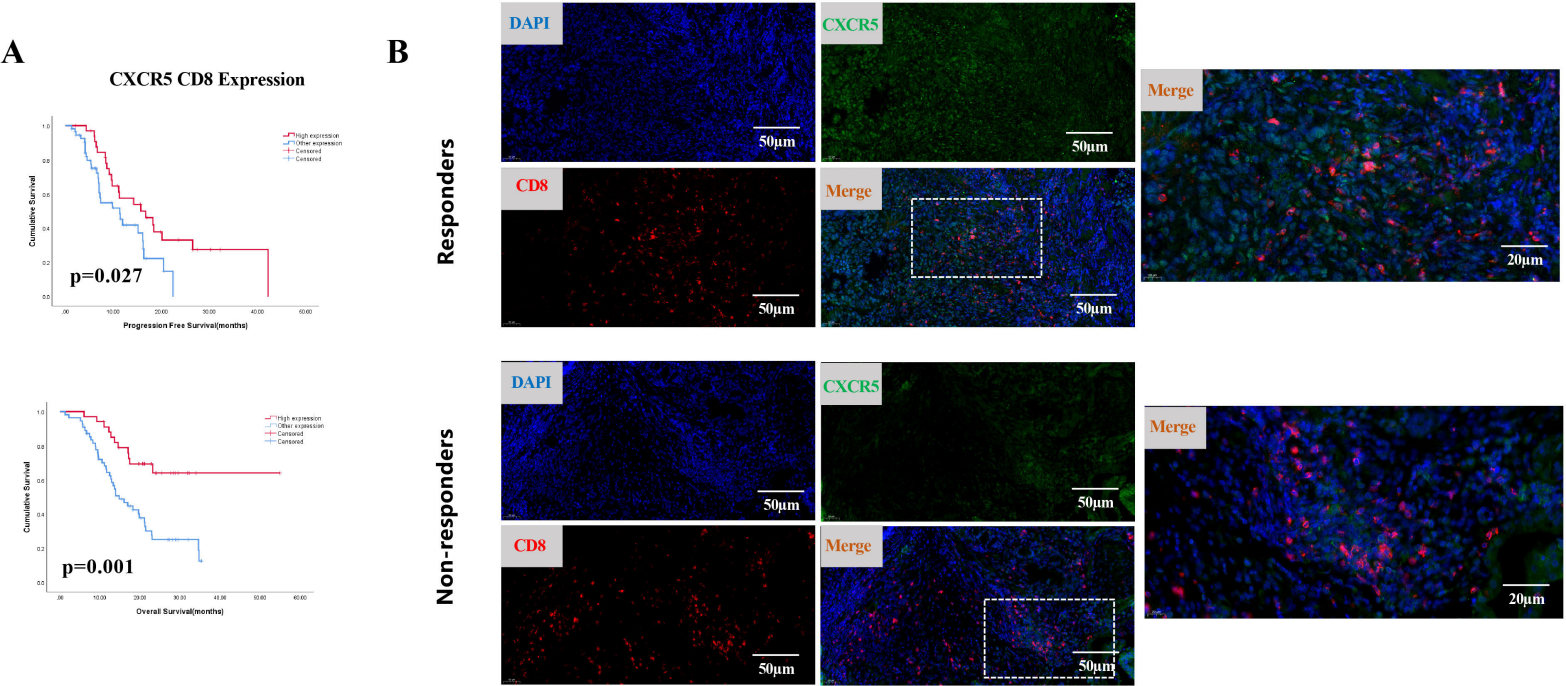
**(A)** Kaplan-Meier survival curves for PFS (top) and OS (bottom) in cohort 1, comparing patients with simultaneous high expression of CXCR5 and CD8 (CXCR5^H^CD8^H^) to other expression groups (*n* = 89; log-rank test, *p*-values shown). **(B)** Representative IF staining of responders and non-responders. Samples were stained for CXCR5 (green), CD8 (red), and DAPI (blue).

CXCL13 is the exclusive ligand for CXCR5. Therefore, we hypothesized that CXCR5+ lymphocytes might participate in CXCL13-mediated immunotherapy responses. To investigate this, we first analyzed the correlation between CXCL13 expression and the presence of CXCR5+ immune cells. Simultaneous high expression of CXCR5 and CD8 was defined as the CXCR5^H^CD8^H^ group, whereas simultaneous high expression of CXCR5 and CD4 was defined as the CXCR5^H^CD4^H^ group. Low expression of both markers or high expression of only one marker was categorized as the “other expression” group. A strong correlation was observed between CXCL13 expression and both CXCR5^H^CD8^H^ and CXCR5^H^CD4^H^ expression. No significant associations were found between CXCR5^H^CD8^H^ or CXCR5^H^CD4^H^ and other clinical characteristics (**Supplementary Table S2**). Moreover, patients with CXCR5^H^CD8^H^ expression demonstrated significantly better PFS (*p* = 0.027) and OS (*p* = 0.001) than those in the other expression group (**Figure 2A**). The same phenomenon was observed in the external databases. As single markers, neither CXCR5 nor CD8 expression was significantly associated with GC survival outcomes (**Supplementary Figure S2**). Patients with CXCR5^H^CD4^H^ expression exhibited improved OS (*p* = 0.044) (**Supplementary Figure S3A**). Intriguingly, double-label immunofluorescence staining revealed that CXCR5-expressing cells colocalized with CD8 and CD4 in CR and PR patients (**Figure 2B**; **Supplementary Figure S3B**).

**Figure 3:**
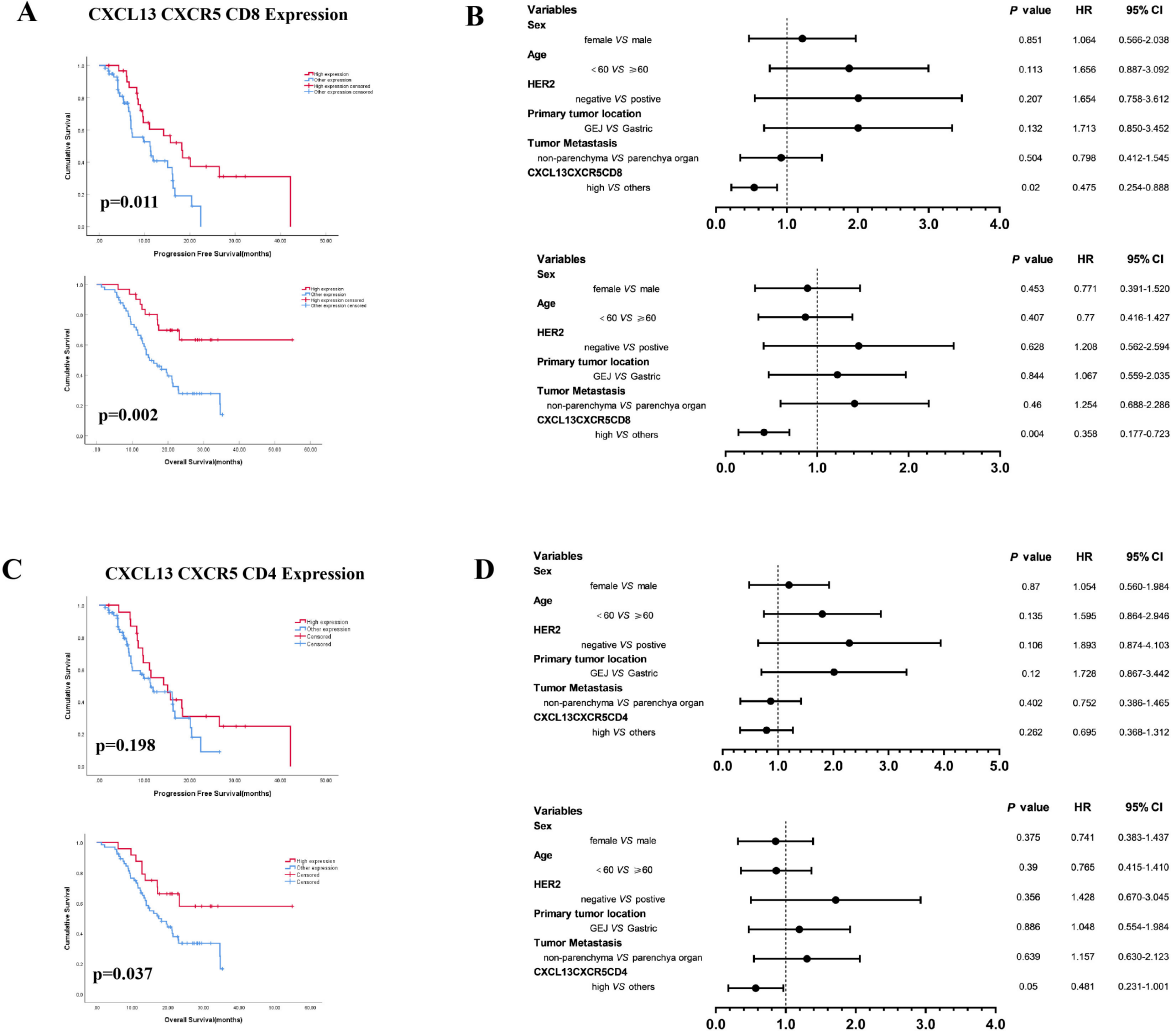
**(A)** Kaplan–Meier survival curves for PFS and OS according to concurrent high expression of CXCL13, CXCR5, and CD8 (CXCL13^H^CXCR5^H^CD8^H^ vs. other expression). **(B)** Multivariate analysis based on clinicopathological characteristics and the expression of CXCL13, CXCR5, and CD8 in cohort 1 patients. **(C)** CXCL13, CXCR5, and CD4 (CXCL13^H^CXCR5^H^CD4^H^ vs. other expression). **(D)** Multivariate analysis based on clinicopathological characteristics and the expression of CXCL13, CXCR5, and CD4 in cohort 1 patients (log-rank test for Kaplan–Meier curves. HR, hazard ratio; CI, confidence interval).

Next, we investigated the prognostic significance of the combined expression of CXCL13, CXCR5, and CD8 or CD4. The results indicated that concurrent high expression of CXCL13, CXCR5, and CD8 (CXCL13^H^CXCR5^H^CD8^H^) predicted superior survival outcomes (**Figure 3A**). Concurrent high expression of these three markers was an independent prognostic factor, correlating with better PFS (HR: 0.475, 95% CI: 0.254–0.888) and OS (HR: 0.358, 95% CI: 0.177–0.723) in cohort 1 patients (**Figure 3B**). Patients with concurrent high expression of CXCL13, CXCR5, and CD4 (CXCL13^H^CXCR5^H^CD4^H^) demonstrated prolonged OS (HR: 0.481, 95% CI: 0.231–1.001) (**Figures 3C, D**).

### CXCR5 expression was correlated with the presence of TLSs in GC patients

**Figure 4:**
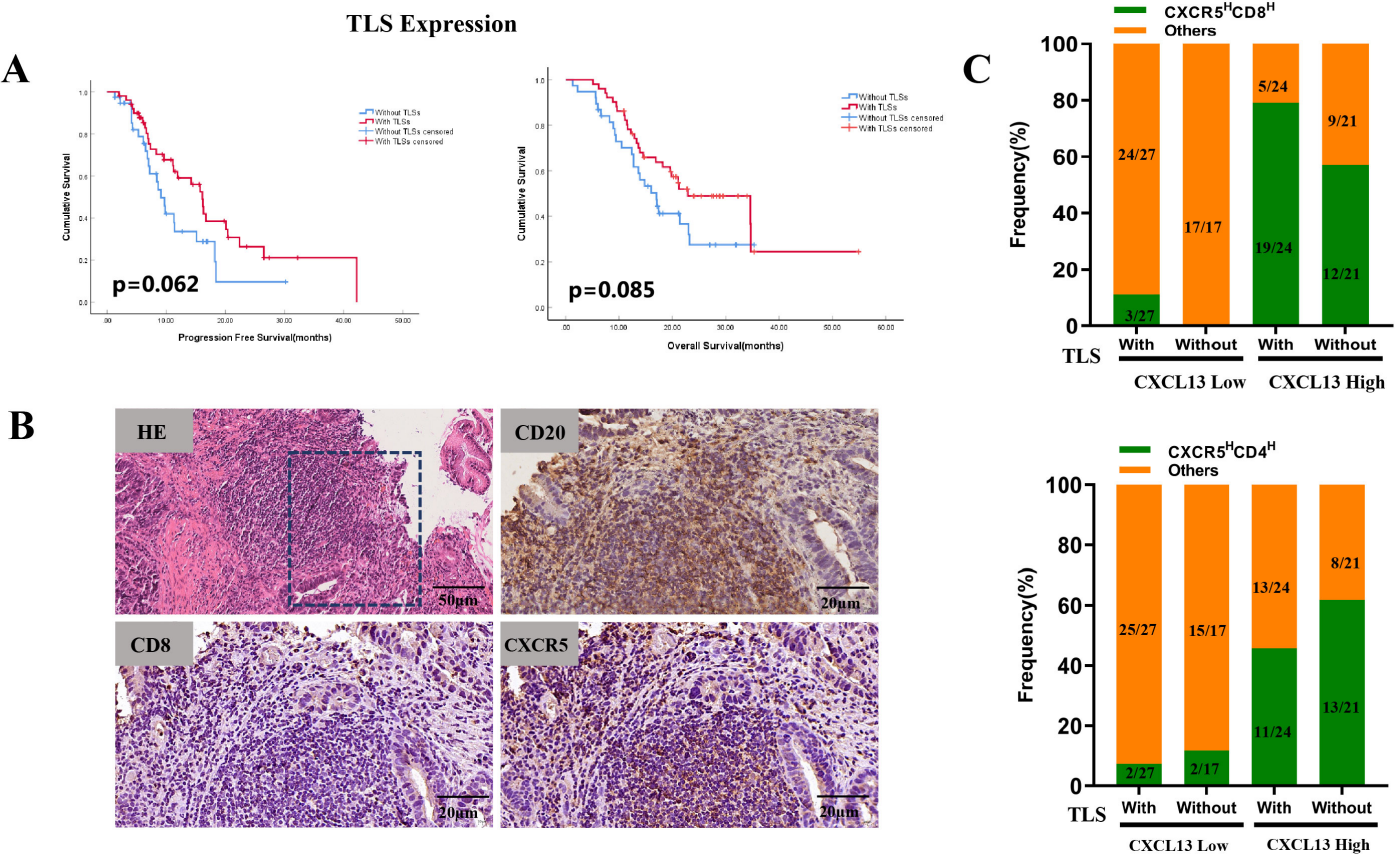
**(A)** Kaplan–Meier survival curves for PFS (left) and OS (right) in cohort 1 patients stratified by the presence of TLSs. **(B)** Representative images of TLSs (H&E staining) and expression of CD20, CD8, and CXCR5 within TLSs. **(C)** Frequencies of CXCR5^H^CD8^H^ and CXCR5^H^CD4^H^ expression were compared to other expression groups, based on TLS presence and CXCL13 expression levels.

Previous studies have demonstrated that TLSs may influence the efficacy of immunotherapy by regulating immune cell infiltration. The CXCL13–CXCR5 axis jointly contributes to the formation of TLSs in malignant melanoma, and the presence of TLSs is associated with increased benefits from ICI treatment (27). In our study, TLSs were identified in 51 of the 88 GC specimens. Survival analysis indicated that the presence of TLSs improved the immunotherapy outcomes. The median PFS was 16.1 months in patients with TLSs compared with 9.1 months in those without TLSs (*p* = 0.062). The median OS was 22.9 months vs. 17.0 months, respectively (*p* = 0.085) (**Figure 4A**). Among the patients with TLSs, 24 exhibited high CXCL13 expression and prolonged PFS (*p* = 0.039) and OS (*p* < 0.001) (**Table 2**). Of these 24 patients, 19 (79.2%) showed concurrent high expression of CXCR5 and CD8 (CXCR5^H^CD8^H^) (**Figure 4C**). IHC results further revealed significant enrichment of CXCR5 within TLSs (**Figure 4B**). In contrast, among patients with low CXCL13 expression, the proportion of CXCR5^H^CD8^H^ was lower, regardless of the presence of TLSs (**Figure 4C**). These findings suggest that the CXCL13–CXCR5 axis may contribute to TLS formation, potentially regulating CD8+ T-cell infiltration and influencing clinical responses to immunotherapy in GC.

**Table 2:** Analysis of the correlation between molecular marker expression with/without TLSs and survival.

Variable	PFS	OS
	*p*-value	*p*-value
With TLSs		
CXCL13 expression		
High vs. low	0.039	< 0.001
CXCR5 expression		
High vs. low	0.457	0.004
Without TLSs		
CXCL13 expression		
High vs. low	0.261	0.601
CXCR5 expression		
High vs. low	0.47	0.539

### CXCL13 enhances the response to anti-PD-1 therapy in a humanized subcutaneous GC mouse model

**Figure 5:**
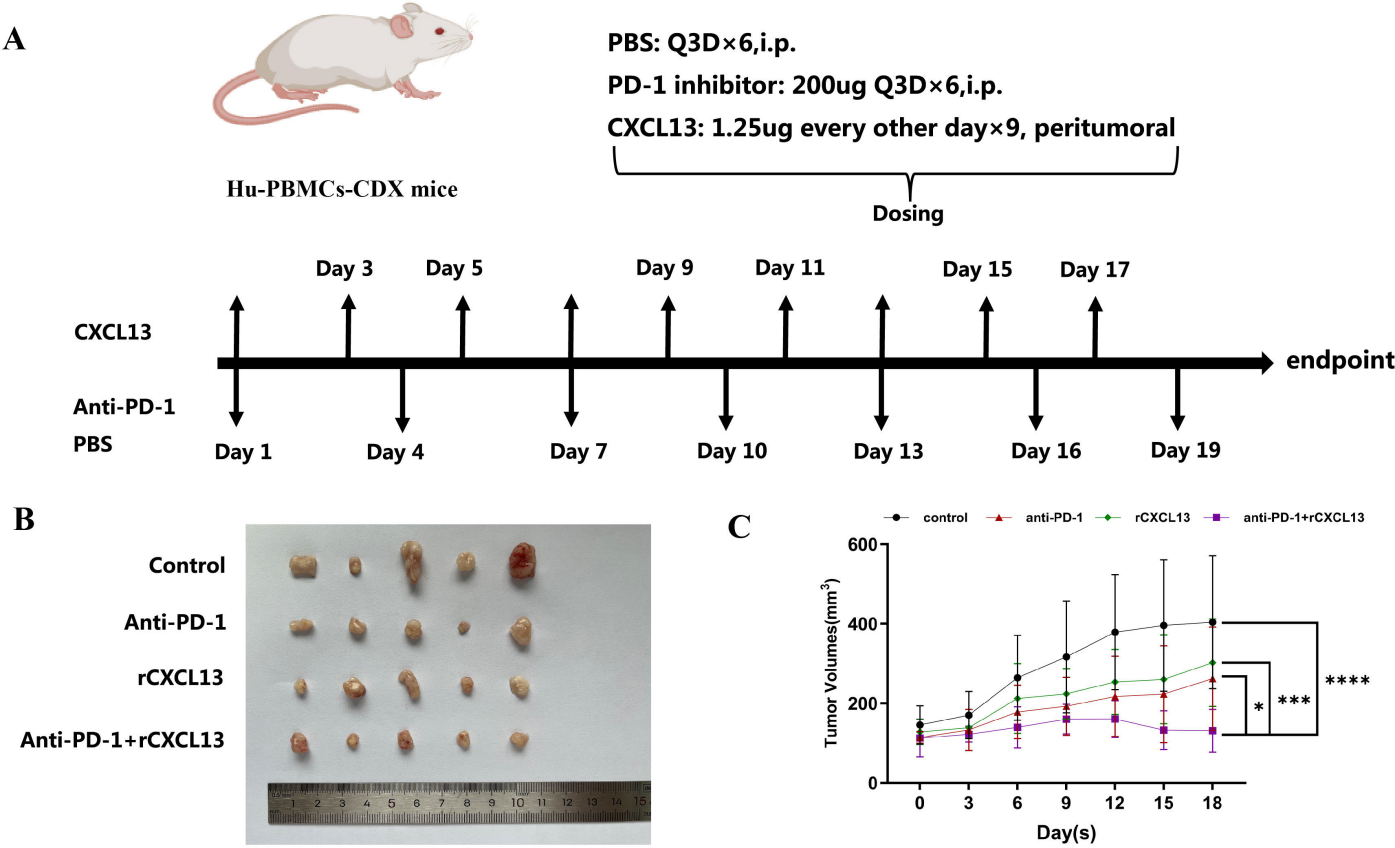
**(A)** Schematic depiction of the animal experiments and treatment administration details. **(B)** Combined treatment with CXCL13 and anti-PD-1 antibody effectively suppressed subcutaneous tumor growth. **(C)** Tumor growth curves comparing different treatment groups. (^*^ *p* < 0.05; ^***^ *p* < 0.001; ^****^ *p* < 0.0001).

**Figure 6:**
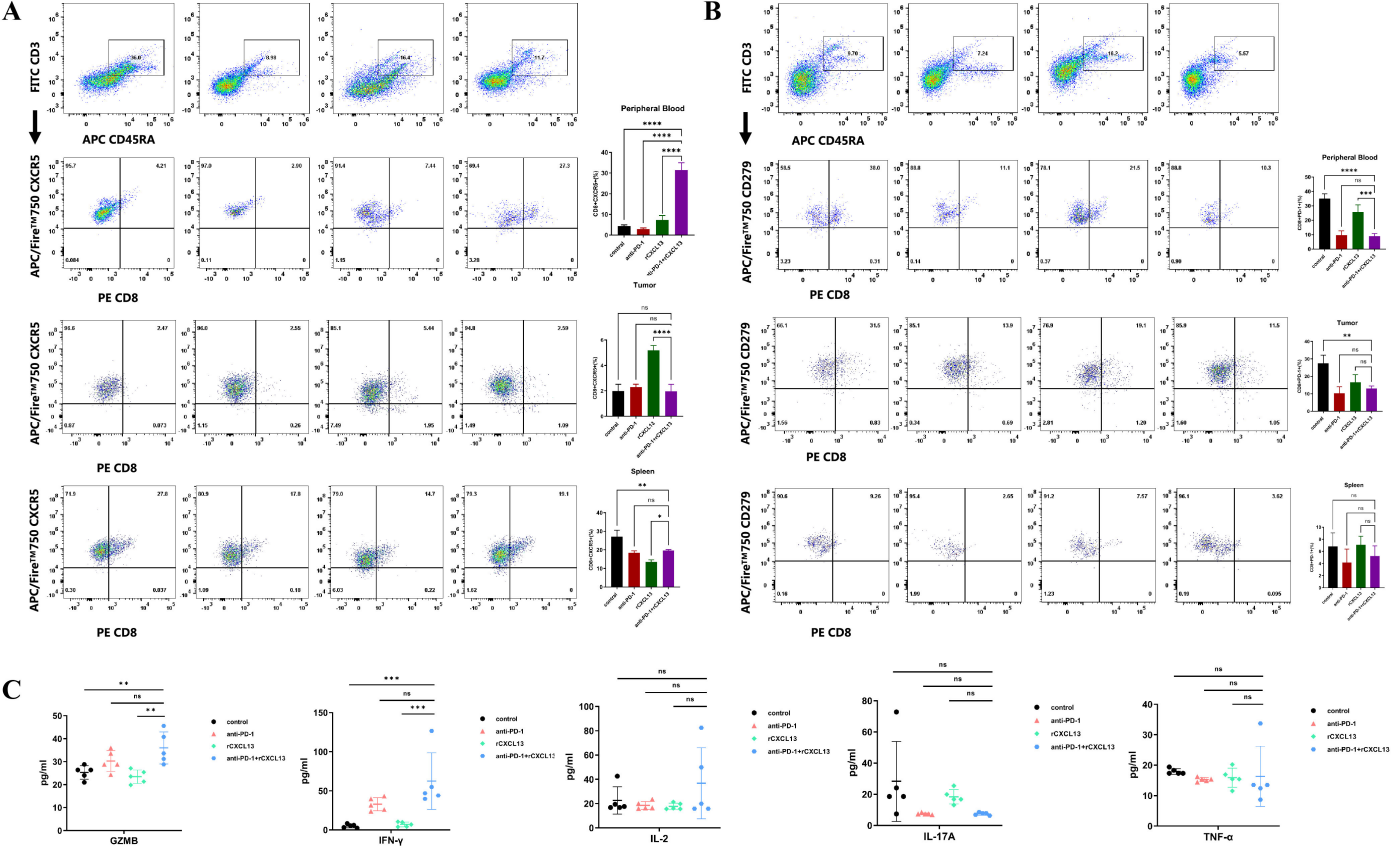
**(A)** Percentage of CXCR5+CD8+ T cells and **(B)** PD-1+CD8+ T cells in peripheral blood, subcutaneous tumor, and spleen across the four indicated treatment groups (*n* = 5 per group). **(C)** Percentage of effector cytokines (GZMB, IFN-γ, IL-2, IL-17A, and TNF-α) in peripheral blood across the four indicated groups (*n* = 5 per group). (Bar plots represent mean ± SD; ^**^ *p* < 0.01; ^***^ *p* < 0.001; ^****^ *p* < 0.0001; ns, not significant).

Given our findings that concurrent high expression of CXCL13, CXCR5, and CD8+ T cells predicts a favorable clinical response to ICIs in patients with GC and that CXCR5+CD8+ T cells may play a pivotal role during ICI treatment, we further investigated the impact of CXCL13 on anti-PD-1 therapy. Xenograft tumor models were established using the GC cell line MKN-28 in immunodeficient NOD/SCID mice, which were humanized via the engraftment of PBMCs (**Figure 5A**). Combination treatment with CXCL13 and anti-PD-1 antibody significantly suppressed tumor growth compared to both the monotherapy and control groups (**Figures 5B, C**). Furthermore, the combination therapy increased the proportion of CXCR5+CD8+ T cells and reduced the ratio of PD-1+CD8+ T cells in the peripheral blood (**Figures 6A, B**). No significant differences in immune cell infiltration were observed in the spleen or tumor tissues across the groups. Additionally, combination treatment significantly promoted the expression of the effector cytokines GZMB and IFN-γ compared to other groups (**Figure 6C**). These findings suggest that CXCL13 may enhance the response to anti-PD-1 antibody therapy by expanding the population of CXCR5+CD8+ T cells.

## Discussion

This study identified the regulatory functions of CXCL13 in immune cell infiltration within the TME of GC patients and highlighted its potential role as a molecular marker for immunotherapy. Elevated CXCL13 expression was associated with prolonged survival. The combined evaluation of CXCL13, CD8, and CXCR5 was confirmed to be an independent prognostic factor in GC patients who received ICI therapy. Previous research has shown that, in EBV-positive GC patients, the clonal expansion of CXCL13+CD8+ T cells is substantially increased in responders after immunotherapy (28). CXCL13 has been identified as a critical regulator of immune cell recruitment and differentiation within the TME in GC patients with signet ring cell carcinoma (29). Additionally, CXCL13 may attract CXCR5+CD8+ T cells to the TME and enhance cytotoxic T-lymphocyte function by regulating the expression of GZMB, TNF-α, IFN-γ, and other cytokines (30). Concordantly, our study also observed significantly elevated GZMB and IFN-γ levels during immunotherapy, which may indicate the activation of CXCR5+CD8+ T cells. These results suggest that CXCL13 plays a vital role in PD-1-based therapies for GC.

CXCL13 influences the efficacy of ICI treatment by modulating immune cell infiltration in various solid cancers. High levels of pre-treatment CXCL13+ T cells are associated with pro-inflammatory macrophage activity and predict favorable responses in patients with triple-negative breast cancer receiving paclitaxel combined with a PD-L1 monoclonal antibody (31). Interactions between follicular helper T cells (CD4 T_FH_), tissue-resident T cells (CD8 T_RM_), and B cells mediated by the CXCL13–CXCR5 axis are crucial for anti-tumor immunity in EGFR-mutated non-small cell lung cancer (NSCLC). Dysregulation of T_FH_-B and T_RM_-B crosstalk may contribute to poor responses to PD-1 blockade in EGFR-mutated NSCLC (32). In ovarian cancer, combination therapy with CXCL13 and an anti-PD-1 antibody inhibited tumor growth in a CXCR5+CD8+ T-cell expansion-dependent manner (23). Similarly, tumor growth in NSCLC was effectively suppressed by combination treatment (26). In bladder cancer, data from the CheckMate-275 and IMvigor210 trials demonstrated that patients with ARID1A mutations and high CXCL13 expression had favorable outcomes following ICI treatment (33).

In addition to exerting anti-tumor functions through immunomodulatory effects on the TME, studies have reported that CXCL13 may directly affect tumor cells. In prostate cancer, activation of the nonclassical NF-κB signaling pathway promotes the secretion of CXCL13 in tumor cells. Elevated CXCL13 secretion, in turn, enhances tumor growth, migration, and invasion through CXCR5 on the tumor cell surface (34). Furthermore, CXCL13 increases the expression of EVT4 in pancreatic ductal adenocarcinoma (PDAC), and EVT4 promotes PDAC invasion and metastasis by binding to CXCR5 on the tumor cell surface (35). These results suggest that CXCL13 may exert a direct non-chemokine effect on tumor cells. However, further confirmation is needed due to inconsistencies between the study findings.

TLSs serve as prognostic and predictive biomarkers (36) and are linked to higher objective responses to PD-1 blockade in various solid tumors. During tumorigenesis, TLSs act as effective sites for tumor-immune interactions within the TME, triggering inflammatory responses by immune cell infiltration (37). In patients with stage IV GC, responders exhibited a higher percentage of TLSs and increased infiltration of CXCL13+CD160+CD8+ T cells following immunochemotherapy (38). Furthermore, upregulation of CXCL13 expression facilitates the recruitment of CXCR5+ B cells and the formation of TLSs. Alternatively, one study found that CXCL13+CD103+CD8+ Trm cells within TLSs were associated with a better response to anti-PD-1 therapy (39). This suggests a significant role for CXCL13-dependent TLS formation in the efficacy of ICI treatment (23, 40). In our study, patients with TLSs showed improved outcomes from immunotherapy. CXCL13 increased the expression of CXCR5 and CD8+ T cells, particularly in patients with TLSs. TLSs are composed of various immune cell types, and the characteristics of these cells are crucial for TLS maturation (41). Mature TLSs are more likely to show significant clinical benefits after ICI treatment (42). Therefore, multiple factors likely contribute to the antitumor immunity of TLSs, and the specific regulatory mechanisms underlying CXCL13-dependent TLS formation warrant further investigation.

This study has several limitations. First, the sample size is relatively small. Second, most of the patients had stage IV diseases, which led to clinically heterogeneous findings. Third, further mechanistic investigation is needed, including additional molecular biology experiments to verify the mechanism by which CXCL13 recruits CXCR5+CD8+ T cells to the TME and promotes the formation of TLSs, as well as the co-expression of the above indicators in GC. Despite these limitations, our findings highlight the clinical predictive value of CXCL13 in GC and suggest that its underlying mechanisms warrant further exploration.

In summary, our offer insights into how CXCL13 promotes the response of GC patients to ICI therapy. We identified CXCL13 as a prognostic marker for GC and demonstrated that its critical role in the antitumor microenvironment is mediated through CXCR5+CD8+ T cells.

## Data Availability

The original contributions presented in the study are included in the article/**Supplementary Material**. Further inquiries can be directed to the corresponding authors.
