# Supplementary material for: High expression of CXCL13 predicts a favorable response to immunotherapy by upregulating CXCR5+CD8+ T-cell infiltration in gastric cancer
Source: Front Immunol. 2025 May 8;16:1551259. doi: 10.3389/fimmu.2025.1551259 (PMC12095368; doi:10.3389/fimmu.2025.1551259)
Supplement: Supplementary file 1 [file DataSheet1.docx]

**Supplementary Figure 1:**

**Figure S1:** (A). Schematic representation of the humanized PBMCs subcutaneous tumor mouse model establishment. (B). PCR analysis of CXCL13 expression in various GC cells.


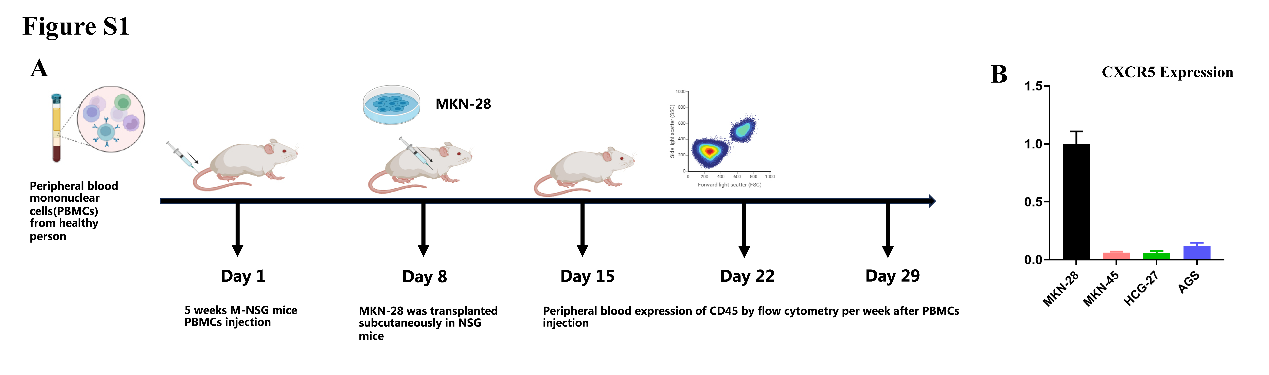


**Supplementary Figure 2:**

**Figure S2:** Survival analysis of CXCR5(left) and CD8(right) the TGCA and GEO datasets.


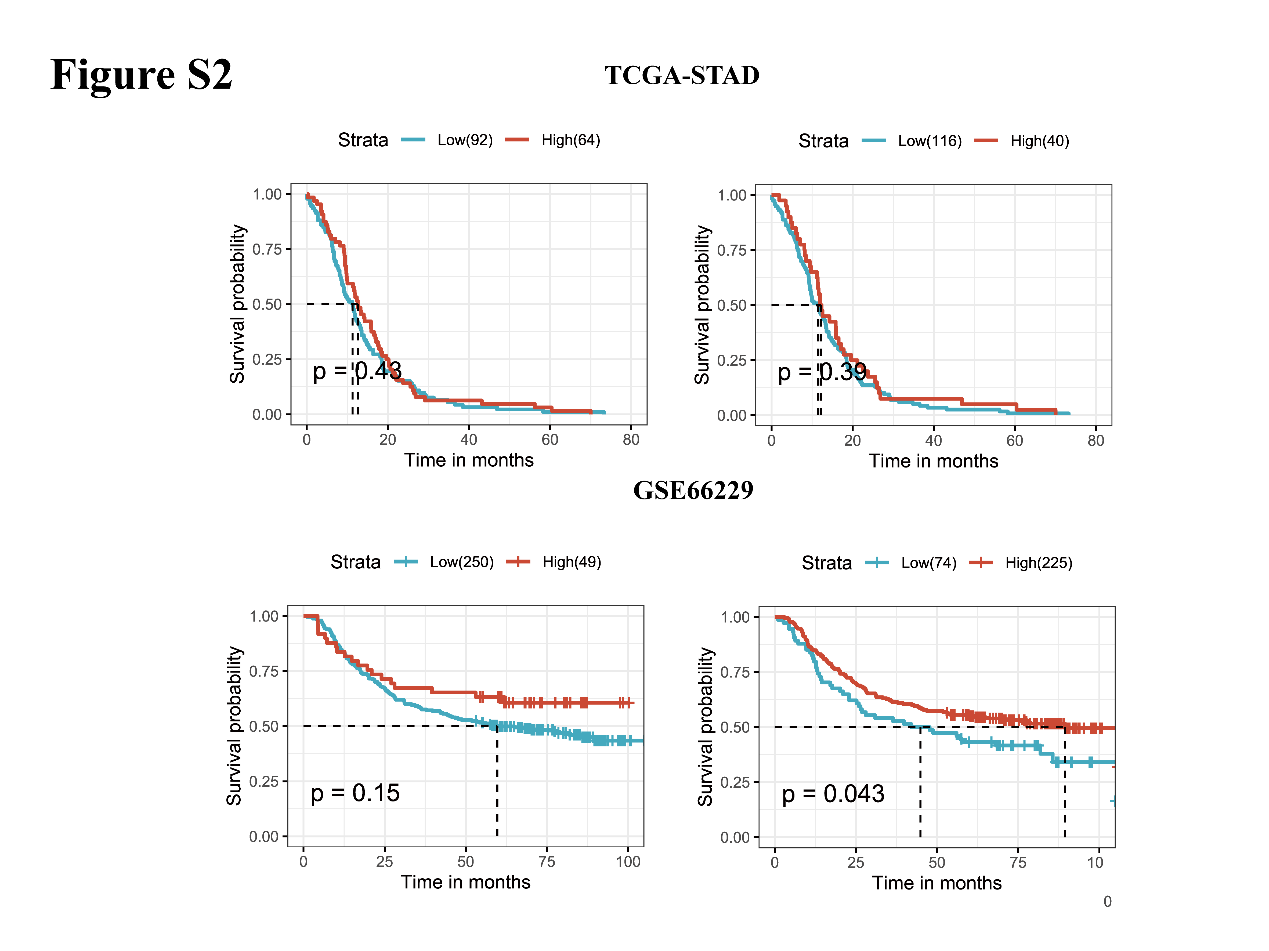


**Supplementary Figure 3:**

**Figure S3:** (A). Kaplan-Meier survival curves for PFS (up) and OS (down) of cohort 1 patients between simultaneous high expression of CXCR5 and CD4(CXCR5^H^CD4^H^) group and other expression patients (n=89; log-rank test and p values are shown). (B). Representative IF staining of responders (up) and non-responders(down). The sample was stained for CXCR5 (green), CD4(red), and DAPI (blue).


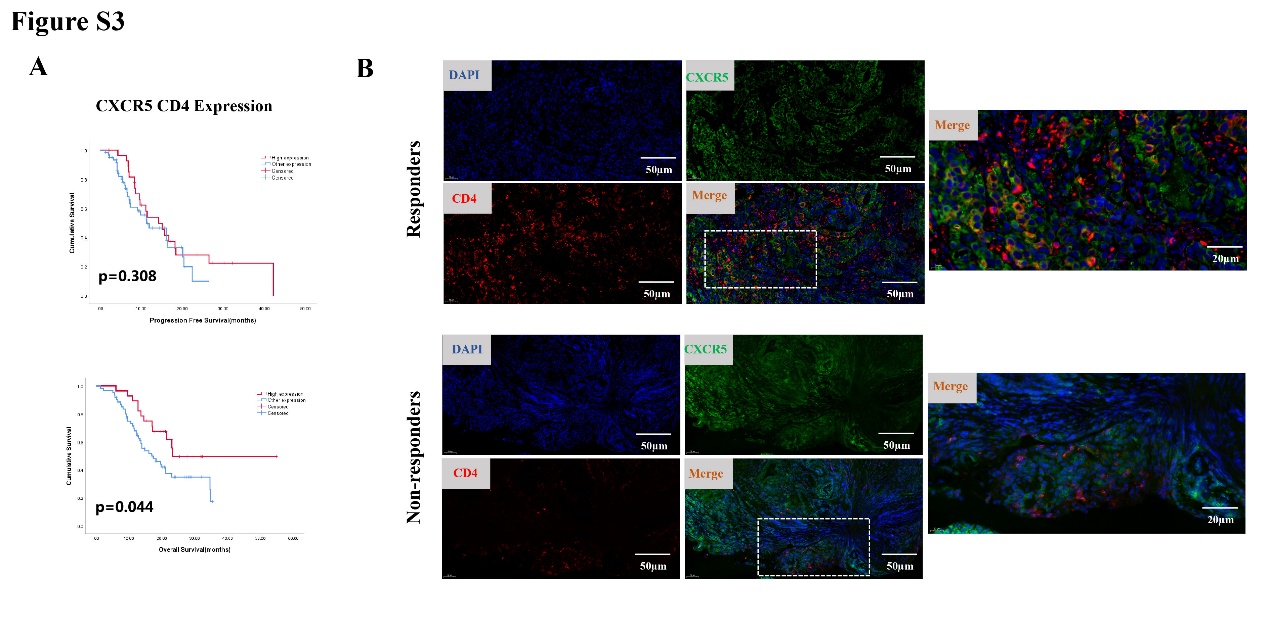


**Supplementary Table 1 (Table S1):**

| **Antibody list of the study** | | | | | | | |
| --- | --- | --- | --- | --- | --- | --- | --- |
| **No.** | **Anti-body name** | **Description** | **Reactivity** | **Manufacturer** | **Catalog No.** | **Dilution** | **Application** |
| 1 | anti-CXCR5 antibody | Rabbit monoclonal | Human | CST | P32302 | 1:200 | Double-staining IHC |
| 2 | anti-CD8 antibody | Rabbit monoclonal | Human | Abcam | ab245118 | working solution | Double-staining IHC |
| 3 | Anti-CD4-antibody | Rabbit monoclonal | Human | Abcam | ab133616 | 1:1200 | Double-staining IHC |
| 4 | Anti-BCA1-antibody | Rabbit monoclonal | Human | Abcam | ab246518 | 1:500 | IHC |
| 5 | Anti-CD4-antibody | Rabbit monoclonal | Human | Abcam | ab133616 | 1:1200 | IHC |
| 6 | anti-CD8 antibody | Rabbit monoclonal | Human | Zhongshan Jinqiao | ZA-0508 | working solution | IHC |
| 7 | anti-CXCR5 antibody | Rabbit monoclonal | Human | CST | P32302 | 1:200 | IHC |
| 8 | CD20 Polyclonal Antibody | Rabbit monoclonal | Human | Invitrogen | PA5-16701 | 1:300 | IHC |
| 9 | APC anti-CD45RA antibody | mouse monoclonal | Human | Biolegend | 983004 | 2ul | FCM |
| 10 | FITC anti-CD3 antibody | mouse monoclonal | Human | Biolegend | 317306 | 2ul | FCM |
| 11 | PE ani-CD4 antibody | mouse monoclonal | Human | Biolegend | 344606 | 2ul | FCM |
| 12 | PE anti-CD8 antibody | mouse monoclonal | Human | Biolegend | 980902 | 2ul | FCM |
| 13 | APC/FireTM750 anti-CD185(CXCR5) antibody | mouse monoclonal | Human | Biolegend | 356946 | 2ul | FCM |
| 14 | APC/FireTM750 anti-CD279(PD-1) antibody | mouse monoclonal | Human | Biolegend | 329953 | 2ul | FCM |
| 15 | PE-anti-CD45 antibody | mouse monoclonal | Human | CST | P08575 | 2ul | FCM |

**Supplementary Table 2 (Table S2):**

| **The association between patient characteristics and biomarkers** | | | | | | |  |
| --- | --- | --- | --- | --- | --- | --- | --- |
|  | **CXCR5CD8 expression** | | | **CXCR5CD4 expression** | | | |
| **Variables** | **High** | **Others** | **p value** | **High** | **Others** | **p value** |  |
| **Age** |  |  |  |  |  |  |  |
| ≥60 | 18 | 33 | 0.513 | 16 | 35 | 0.983 |  |
| ＜60 | 16 | 22 |  | 12 | 26 |  |  |
| **Sex** |  |  |  |  |  |  |  |
| Male | 24 | 37 | 0.743 | 19 | 42 | 0.925 |  |
| Female | 10 | 18 |  | 9 | 19 |  |  |
| **HER-2 status** |  |  |  |  |  |  |  |
| IHC 3+/FISH amplification | 12 | 10 | 0.069 | 7 | 15 | 0.967 |  |
| IHC 0-2+/FISH non-amplification+UK | 22 | 45 |  | 21 | 46 |  |  |
| **Primary tumor location** |  |  |  |  |  |  |  |
| Gastro-oesophagal junction | 9 | 14 | 0.915 | 7 | 16 | 0.902 |  |
| Gastric | 25 | 41 |  | 21 | 45 |  |  |
| **Tumor Metastasis** |  |  |  |  |  |  |  |
| Parenchyma organ | 18 | 25 | 0.492 | 15 | 28 | 0.501 |  |
| Non-parenchyma organ | 16 | 30 |  | 13 | 33 |  |  |
| **CXCL13 expression** |  |  |  |  |  |  |  |
| High | 31 | 14 | ＜0.0001 | 24 | 21 | 0.001 |  |
| Low | 4 | 40 |  | 4 | 40 |  |  |
| **Efficacy** |  |  |  |  |  |  |  |
| CR+PR | 19 | 12 | 0.001 | 14 | 17 | 0.025 |  |
| SD+PD+UK | 15 | 43 |  | 14 | 44 |  |  |
